# Quantitative Measurement of the Kinase Activity of Wildtype ALPK1 and Disease-Causing ALPK1 Mutants Using Cell-Free Radiometric Phosphorylation Assays

**DOI:** 10.21769/BioProtoc.5124

**Published:** 2024-11-20

**Authors:** Tom Snelling

**Affiliations:** MRC Protein Phosphorylation and Ubiquitylation Unit, School of Life Sciences, University of Dundee, Scotland, UK

**Keywords:** ALPK1, Nucleotide sugar, ADP-heptose, ROSAH, Spiradenoma, Phosphorylation, Protein kinase

## Abstract

ALPK1 is an atypical protein kinase that is activated during bacterial infection by ADP-heptose and phosphorylates TIFA to activate a cell signaling pathway. In contrast, specific mutations in ALPK1 allow it to also be activated by endogenous human nucleotide sugars such as UDP-mannose, leading to the phosphorylation of TIFA in the absence of infection. This protocol describes a quantitative, cell-free phosphorylation assay that can directly measure the catalytic activity of wildtype and disease-causing ALPK1 in the presence of different nucleotide sugars. In this method, overexpressed ALPK1 is first immunoprecipitated from the extracts of ALPK1 knockout HEK-Blue cells transfected with plasmids encoding either FLAG-tagged wildtype or mutant ALPK1, and then subjected to a radioactive phosphorylation assay in which the phosphorylation of purified GST-tagged TIFA by ALPK1 is quantified by measuring the incorporation of radioactivity derived from radiolabeled ATP.

Key features

• Quantitative measurement of protein kinase activity of wildtype and mutant ALPK1 in the presence or absence of different nucleotide sugars such as ADP-heptose and UDP-mannose.

• Cell-free experimental setup overcoming the challenge of distinguishing constitutive activity and activation by endogenous mammalian metabolites in cell-based assays.

• Requires approximately 50 µg of cell extract protein/reaction, allowing up to 150 assays to be performed from an extract prepared from a single 15 cm dish of transfected cells.

## Background

Alpha-protein kinase 1 (ALPK1) is an atypical protein kinase that is activated by the binding of the bacterial metabolite ADP-heptose to its N-terminal domain [1]. This allows ALPK1 to phosphorylate TIFA (TRAF-interacting protein with forkhead-associated domain), which in turn triggers its polymerization into TIFAsomes that initiate signaling events that lead to the activation of transcription factors such as NF-κB and AP-1 [1,2] (see [3] for a schematic overview of the ADP-heptose signaling pathway).

Mutations in ALPK1 are the cause of at least two human diseases. The Thr237Met, Tyr254Cys, and Ser277Phe mutations cause ROSAH syndrome (retinal dystrophy, optic nerve edema, splenomegaly, anhidrosis, and migraine headache), which is an autosomal dominant genetic disorder [4–6], whereas the ALPK1[V1092A] mutation is a driver of spiradenoma, a rare type of hair follicle tumor that can transform into a malignant form called spiradenocarcinoma, which is invariably fatal [7]. These mutations allow ALPK1 to be activated by endogenous mammalian nucleotide sugars, such as UDP-mannose and ADP-ribose, in addition to bacterial ADP-heptose, leading to pathological signaling in the absence of bacterial infection [3,6].

This protocol describes a cell-free phosphorylation assay that can be used to quantify the protein kinase activity of wildtype and mutant forms of ALPK1 in the presence or absence of different nucleotide sugars. Existing methods for measuring the activity of ALPK1 mutants have relied on measuring their activity within cells, but the complexity of the intracellular environment makes it challenging to identify specific activators. Furthermore, these cell-based methods rely on indirect measurements of ALPK1 activity after many hours, such as the activation of transcription factors or the secretion of cytokines or chemokines. In contrast, this phosphorylation assay has the advantage of enabling rapid and quantitative measurements of ALPK1 activity by directly measuring its ability to phosphorylate TIFA in a cell-free system. Additionally, this assay is ideal for validating potential ALPK1 inhibitors by studying their potency and selectivity for targeting wildtype or mutant forms of this protein kinase.

## Materials and reagents

**Biological materials**

Cells should be cultured by incubation at 37 °C with 5% CO_2_ and tested regularly for mycoplasma using a MycoAlert Mycoplasma Detection Kit (Lonza, catalog number: LT07-318). The cells should be passaged once confluent at a ratio of 1:10 (v/v), and not used beyond 30 passages.

1. ALPK1 knockout (KO) HEK-Blue cells (InvivoGen, #hkb-koalpk)

**General reagents**

Storage conditions are given in parentheses unless it is room temperature.

1. ADP-L-heptose triethylammonium salt (InvivoGen, catalog number: tlrl-adph-l) (-80 °C)
*Note: Resuspend 250 µg in 3,470 µL of reaction buffer (see Recipes) to generate a 0.1 mM stock.*
2. UDP-α-D-mannose triethylammonium salt (synthesized in-house, available upon request) (-80 °C)
*Note: Resuspend 2 mg in 3,280 µL of reaction buffer to generate a 1 mM stock.*
3. ADP-D-ribose sodium salt (Sigma-Aldrich, catalog number: C7344) (-80 °C)
*Note: Resuspend 2 mg in 3,580 µL of reaction buffer to generate a 1 mM stock.*
4. 3,000 Ci/mmol [γ^32^P]ATP (Revvity, catalog number: BLU002A001MC) (-20 °C)
**Caution:** [γ^32^P]ATP must be handled according to radioactivity safety regulations. Exposure to radiation must be minimized by working behind a plexiglass shield and storing samples in plexiglass boxes.
5. Dulbecco’s modified Eagle’s medium (DMEM) (Gibco, catalog number: 11960-085) (4 °C)
6. 200 mM L-Glutamine (Gibco, catalog number: 25030024) (-20 °C)
7. Penicillin-Streptomycin 100× stock (Gibco, catalog number: 15140122) (-20 °C)
8. OptiMEM I reduced serum medium (Gibco, catalog number: 31985062) (4 °C)
9. Lipofectamine 2000 (Thermo Fisher, catalog number: 11668019) (4 °C)
10. Trypan Blue solution (Gibco, catalog number: 11538886)
11. Anti-FLAG M2 affinity resin (Millipore, catalog number: A2220)
12. Phosphate-buffered saline (PBS) (Gibco, catalog number: 10010023) (4 °C)
13. 10 mM ATP (Thermo Fisher, catalog number: PV3227) (-20 °C)
*Note: This commercial stock solution does not contain magnesium ions.*
14. 5 M NaCl (Sigma-Aldrich, catalog number: S6546)
15. 1 M DTT (Thermo Fisher, catalog number: P2325) (-20 °C)
*Note: Prepare 0.1 mL single-use aliquots.*
16. 1 M Tris-HCl (pH 7.5) (Thermo Fisher, catalog number: 15567027)
17. 0.5 M EGTA (pH 8.0) (Thermo Fisher, catalog number: J60767.AD)
18. 0.5 M EDTA (pH 8.0) (Sigma-Aldrich, catalog number: 03690)
19. 10% (v/v) Triton X-100 (Sigma-Aldrich, catalog number: 93443) (4 °C)
20. Fetal bovine serum (FBS) (Sigma-Aldrich, catalog number: F7524) (-20 °C)
21. Sucrose powder (VWR, catalog number: 27480.360)
22. InstantBlue protein stain (Abcam, catalog number: ab119211) (4 °C)
23. MOPS SDS running buffer 20× stock (Formedium, catalog number: SDS5000)
24. NuPAGE LDS sample buffer 4× stock (Thermo Fisher, catalog number: NP0007)
25. Precision Plus protein standards (Bio-Rad, catalog number: 1610374) (-20 °C)
26. 2-Mercaptoethanol (Sigma-Aldrich, catalog number: M6250)
27. Bovine serum albumin (BSA) (Sigma-Aldrich, catalog number: 810533) (4 °C)
28. Bradford Protein Assay Kit (Thermo Fisher, catalog number: 23200) (4 °C)
29. 1 M Magnesium acetate (MgAc) (Sigma-Aldrich, catalog number: 63052)
30. Trypsin-EDTA solution (Gibco, catalog number: 25200056) (4 °C)
31. Complete EDTA-free protease inhibitor cocktail (Roche, catalog number: 11873580001) (4 °C)

**Purified proteins**

TIFA was purified from bacteria as a GST fusion and is available upon request via the MRC PPU Reagents and Services website (https://mrcppureagents.dundee.ac.uk).

1. GST-TIFA[2-184] (MRC PPU Reagents and Services, catalog number: DU4241) (-80 °C)
*Note: Dialyzed against reaction buffer and stored at 1 mg/mL in single-use aliquots.*

**Plasmids**

These plasmids encode FLAG-ALPK1 or FLAG alone (empty vector control) under a CMV promoter and were purified using NucleoBond Xtra Midi Endotoxin-Free kits (Macherey-Nagel, catalog number: 740420). The yield was ~0.5 mg, resuspended to 0.5 mg/mL in endotoxin-free water. They are stored at -20 °C.

1. pcDNA5-FRT/TO-FLAG-ALPK1 (MRC PPU Reagents and Services, catalog number: DU65668)
2. pcDNA5-FRT-TO-FLAG-ALPK1[S277F] (MRC PPU Reagents and Services, catalog number: DU71952)
3. pcDNA5-FRT-TO-FLAG (MRC PPU Reagents and Services, catalog number: DU41457)

**Solutions**

1. Culture media (see Recipes)
2. Lysis buffer (see Recipes)
3. Wash buffer (see Recipes)
4. Salt wash buffer (see Recipes)
5. Reaction buffer (see Recipes)
6. Radioactive ATP solution (see Recipes)

**Recipes**

1. **Culture media (1 bottle)**
  **Table:** 

Reagent	Final concentration	Amount to add
DMEM	Not applicable	500 mL
FBS	10% (v/v)	50 mL
20 mM L-glutamine	2 mM	5.6 mL
Penicillin-Streptomycin 100×	1×	5.6 mL
  Store at 4 °C.
2. **Lysis buffer (50 mL)**
  **Table:** 

Reagent	Final concentration	Amount to add
1M Tris-HCl (pH 7.5)	50 mM	2.5 mL
Sucrose powder	270 mM	4.6 g
10% (v/v) Triton X-100	1% (v/v)	5 mL
0.5 M EDTA (pH 8.0)	1 mM	100 µL
0.5 M EGTA (pH 8.0)	1 mM	100 µL
1 M DTT	2 mM	100 µL
Protease inhibitor cocktail	1×	1 tablet
Water	Not applicable	42.2 mL
  Use immediately.
3. **Wash buffer (1 L)**
  **Table:** 

Reagent	Final concentration	Amount to add
1 M Tris-HCl (pH 7.5)	50 mM	50 mL
10% (v/v) Triton X-100	0.1% (v/v)	10 mL
1 M DTT	2 mM	2 mL
Water	Not applicable	938 mL
  Store at 4 °C. Use within 1 month.
4. **Salt wash buffer (1 L)**
  **Table:** 

Reagent	Final concentration	Amount to add
1 M Tris-HCl (pH 7.5)	50 mM	50 mL
10% (v/v) Triton X-100	0.1% (v/v)	10 mL
5 M NaCl	0.5 M	100 mL
1 M DTT	2 mM	2 mL
Water	Not applicable	838 mL
  Store at 4 °C. Use within 1 month.
5. **Reaction buffer (1 L)**
  **Table:** 

Reagent	Final concentration	Amount to add
1 M Tris-HCl (pH 7.5)	50 mM	50 mL
0.5 M EGTA (pH 8.0)	1 mM	2 mL
1 M DTT	2 mM	2 mL
1 M MgAc	10 mM	10 mL
Water	Not applicable	937.6 mL
  Store at 4 °C. Use within 1 month.
6. **Radioactive ATP solution (150 µL) (sufficient for 60 kinase reactions)**
  **Table:** 

Reagent	Final concentration	Amount to add
Undiluted [γ^32^P]ATP	Not applicable	5–30 µL*
10 mM ATP	1 mM	15 µL
Reaction buffer (recipe 5)	Not applicable	105–130 µL
  Use immediately.
  *The amount of undiluted [γ^32^P]ATP added to the radioactive ATP solution should be calculated based on the activity reference date to achieve a specific activity of approximately 500 cpm per pmol of ATP for each experiment.

**Laboratory supplies**

1. 15 cm Nunc cell culture dishes (Thermo Fisher, catalog number: 168381)
2. 15 and 50 mL conical centrifuge tubes (Greiner, catalog numbers: 188271 and 227261)
3. 250 mL and 1 L Duran bottles (Thermo Fisher, catalog numbers: FB-800-250 and FB-800-1000)
4. Serological pipettes (Thermo Fisher, catalog number: 10710810)
5. Safe-lock 1.5 mL microcentrifuge tubes (Eppendorf, catalog number: 30123611)
*Note: Safe-lock tubes are highly recommended to avoid losing the sample during end-to-end rotation.*
6. Cellometer counting chamber (Nexcelom, catalog number: 11522186)
7. Spin-X 0.22 μm columns (Costar, catalog number: 8161)
8. NuPAGE Bis-Tris 4%–12% 20-well gels (Thermo Fisher, catalog number: WG1402BOX)
9. 80-well cooling chamber for 1.5 mL tubes (Diversified Biotech, catalog number: CHAM-8000)
10. Swann-Morton number 22 disposable scalpels (Scientific Laboratory Supplies, catalog number: INS4767)

## Equipment

The equipment mentioned below are standard laboratory items, and alternatives can be used in all cases.

1. Cellometer Auto 2000 (Nexcelom Bioscience)
2. Pipetman 4-Pipette Kit (Gilson, catalog number: F167360)
3. Stripettor Ultra Pipet Controller (Corning, catalog number: 4099)
4. Thermomixer (Eppendorf, model: Comfort 5355)
5. Cell culture incubator (Binder, model: CB150)
6. Cell culture hood (Conditioned Air Solutions, model: BioMAT 2-SF)
7. Liquid scintillation counter (Revvity, model: Tri-Carb 4910 TR)
8. Geiger counter
9. Plexiglas benchtop shield
10. Allegra X-12 benchtop centrifuge (Beckman Coulter, catalog number: 392474)
11. Benchtop microcentrifuge (Fisherbrand, model: Micro STAR 17)
12. Refrigerated benchtop microcentrifuge (Fisherbrand, model: Accuspin Micro 17)
13. Dry block heater (Grant, model: QBT2)
14. NuPAGE XCell SureLock Midi-Cell system (Invitrogen)
15. Plate reader (BioTek, model: Epoch)

## Software and datasets

1. Image Lab (BioRad, Version 6.0.1)

## Procedure

Before starting this procedure, two confluent 15 cm dishes of ALPK1 KO HEK-Blue cells, no higher than passage 30, are required. This protocol describes how to compare wildtype ALPK1 and the ALPK1[S277F] mutant, but the experiment can also be scaled up to include additional conditions as required.

- **Transient expression of FLAG-tagged ALPK1 constructs in ALPK1 KO HEK-Blue cells (Days 1–2)**
  All steps in section A should be performed in a cell culture hood and the cells should be cultured at 37 °C with 5% CO_2_.
**Plate 3 × 15 cm dishes with 15 million ALPK1 KO HEK-Blue cells each, which will be transfected with empty vector (dish 1) or plasmid encoding FLAG-ALPK1 (dish 2) or FLAG-ALPK1[S277F] (dish 3):**
  1. In the afternoon, aspirate the culture media from two confluent 15 cm dishes of cells and replace with 10 mL of PBS using a serological pipette.
  2. Aspirate the PBS and add 3 mL of trypsin-EDTA solution to each dish. Return the dishes to the incubator until the cells have detached, which should take 2–3 min for this cell line.
  3. Add 15 mL of culture media to each plate and pipette up and down using a serological pipette until a single-cell suspension has been produced. Combine the cell suspensions into a 50 mL canonical centrifuge tube.
  4. Remove 20 µL of the cell suspension and dilute with 80 µL of culture media. Mix 20 µL of the diluted cell suspension with 20 µL of trypan blue solution and count the number of cells using standard methods, ensuring that the cell viability is at least 90%.
  5. Plate 15 million ALPK1 KO HEK-Blue cells into each of the 3 × 15 cm dishes and add media up to a total volume of 20 mL. Ensure that cells are evenly distributed by moving plates in a figure-of-8 motion prior to returning them to the incubator for 18 h.
*Note: Do not change to antibiotic-free culture media for transfection in this protocol.*
**Transfection of ALPK1 KO HEK-Blue cells with FLAG-tagged ALPK1 constructs:**
  6. After 18 h, confirm that the confluency of the ALPK1 KO HEK-Blue cells is approximately 90%.
  7. For each plate to be transfected, add 150 µL of lipofectamine 2000 to 600 µL of OptiMEM in a 1.5 mL microcentrifuge tube (i.e., prepare 3 tubes in this example). Invert five times to mix. In this example, the plates will be transfected with either empty vector or plasmid encoding either FLAG-ALPK1 or FLAG-ALPK1[S277F] (i.e., 3 dishes).
  8. Dilute 60 µg of each plasmid in 600 µL of OptiMEM in 1.5 microcentrifuge tubes. Invert five times to mix.
  9. Add the diluted lipofectamine 2000 to each diluted plasmid. Invert five times to mix and incubate for 10 min at room temperature.
  10. Add each solution dropwise to the relevant dish of cells and return them to the incubator.
  11. After 4 h, carefully aspirate the culture media and add 15 mL of fresh culture media by pipetting slowly against the side of the plate to minimize cell detachment. This step removes the DNA-lipid complexes, which is observed to minimize toxicity. Return the plates to the incubator for 20 h.
- **Preparation and normalization of cell extracts from transfected ALPK1 KO HEK-Blue cells (Day 3)**
**Preparation of cell extracts from HEK-Blue cells transfected with different plasmids:**
*Note: Cell extracts must not be snap-frozen until the end of Section B, since additional freeze-thaw cycles have been observed to lead to a significant reduction in the activity of ALPK1 from cell extracts.*
  1. Twenty-four hours post-transfection, use a 15 mL serological pipette to pipette the culture media up and down until the cells are detached (trypsinization is not required). Transfer the cell suspensions into 15 mL canonical centrifuge tubes.
  2. Centrifuge the tubes at 800× *g* for 5 min at room temperature to pellet the cells. Aspirate the supernatant, add 15 mL of room-temperature PBS to the pellet (do not resuspend), and repeat the centrifugation and aspiration steps for a total of two PBS washes.
  3. After the final PBS wash, ensure that all residual PBS is aspirated and place the cell pellets on ice. All subsequent steps are performed on ice, outside of a sterile cell culture hood, ensure that all residual PBS is aspirated and place the cell pellets on ice.
  4. Add 1 mL of ice-cold lysis buffer to each cell pellet, pipetting up and down until a homogenous suspension is formed.
  5. Transfer each cell lysate to a pre-chilled 1.5 microcentrifuge tube on ice, centrifuge at 18,000× *g* for 20 min at 4 °C, and transfer each supernatant (cell extract) to a new 1.5 mL microcentrifuge tube on ice.
  6. Use the Bradford protein assay kit to determine the protein concentration in each cell extract. Transfer 5 µL of each cell extract to a 1.5 mL microcentrifuge tube and dilute 1:5 (v/v) by addition of 20 µL of water. Transfer 5 µL of each diluted extract in triplicate to a 96-well plate and 5 µL in triplicate of protein standards containing 2.0, 1.0, 0.75, 0.5, 0.25, 0.125, and 0.0625 mg/mL of BSA in water. Add 195 µL of Bradford reagent and measure the absorbance at 595 nm using a microplate reader. Calculate the protein concentration for each cell extract by interpolation of the BSA standard curve, considering the dilution factor.
  7. Dilute the cell extracts to a final concentration of 2 mg/mL protein using ice-cold lysis buffer in a 15 mL conical centrifuge tube on ice. Leave the samples at 4 °C while performing the steps described below.
**Normalization of cell extracts based on relative ALPK1 expression levels:**
  8. An aliquot of each cell extract (0.2 mg of protein) will be immunoprecipitated using 15 µL of resin to determine the relative levels of each ALPK1 protein. Prepare 55 µL of resin as detailed below, sufficient for three samples plus 20% extra.
  9. Add 110 µL of anti-FLAG M2 affinity resin slurry (50% resin by volume, i.e., 55 µL of resin) to a 1.5 mL microcentrifuge tube on ice using a pipette tip where the narrow end has been trimmed using a scalpel.
  10. Centrifuge the slurry at 2,000× *g* for 1 min at 4 °C to pellet the resin. Aspirate the supernatant and add 1 mL of ice-cold lysis buffer. Ensure that the resin is thoroughly resuspended by inverting five times. Repeat the centrifugation and wash steps twice.
  11. After the final wash step, resuspend the beads in 945 µL of ice-cold lysis buffer and invert five times to ensure that the resin is thoroughly resuspended. Use a trimmed pipette tip to add 270 µL of this slurry to 3 × 1.5 mL microcentrifuge tubes (i.e., 15 µL of resin per tube).
  12. Add 100 µL of ice-cold cell extract to each of the tubes (i.e., 0.2 mg). Incubate at 4 °C for 1 h on a rotating wheel to immunoprecipitate the FLAG-tagged ALPK1 from each cell extract.
  13. Centrifuge the samples at 2,000× *g* for 1 min at 4 °C to pellet the resin, carefully aspirate the supernatant, and resuspend the resin in 1 mL of ice-cold salt wash buffer.
  14. Repeat the centrifugation and wash steps a further two times with ice-cold salt wash buffer (for a total of three washes with this buffer), followed twice with ice-cold wash buffer to remove NaCl prior to SDS-PAGE.
  15. After the final wash, carefully aspirate all residual wash buffer, such that the resin is dry. All following steps in this section are performed at room temperature.
  16. Resuspend the resin in 20 µL of LDS sample buffer 1× with 2.5% (v/v) 2-mercapthetanol and heat for 5 min at 75 °C. This solution is prepared by combining 20 µL of LDS sample buffer 4×, 58 µL of water, and 2 µL of 2-Mercaptoethanol.
  17. Centrifuge the samples at 13,000× *g* for 30 s and transfer supernatants to Spin-X columns to remove resin.
  18. Centrifuge the Spin-X columns at 13,000× *g* for 30 s and analyze 10 µL of the eluent (i.e., 50%) by SDS-PAGE alongside 5 µL of the protein ladder (Precision Plus Protein Standards) (see Figure 1A).
  19. Stain the SDS-PAGE gel for 30 min with InstantBlue and destain for 1 h in water with regular changes.
  20. Image the SDS-PAGE gel and quantify the intensity of each FLAG-ALPK1 mutant relative to the WT, for example using Image Lab software.
  21. Calculate the dilution required to normalize cell extracts based on the expression of each ALPK1 in each lysate. In other words, dilute each cell extract to match the concentration of ALPK1 present in the sample containing the lowest amount of ALPK1 (i.e., if the concentration of ALPK1 in a given sample is twice as high as that of the lowest, dilute it in an equal volume). Perform the dilutions using lysate prepared from cells transfected with empty vector plasmid, such that the final protein concentration of each lysate remains unchanged.
  22. In this example, FLAG-ALPK1 and FLAG-ALPK1[S277F] were expressed successfully, leading to a band at approximately 140 kDa that was absent from the empty vector control (Figure 1A). These two forms of ALPK1 were expressed equally well, and normalization was therefore not needed.
    **Figure 1.**
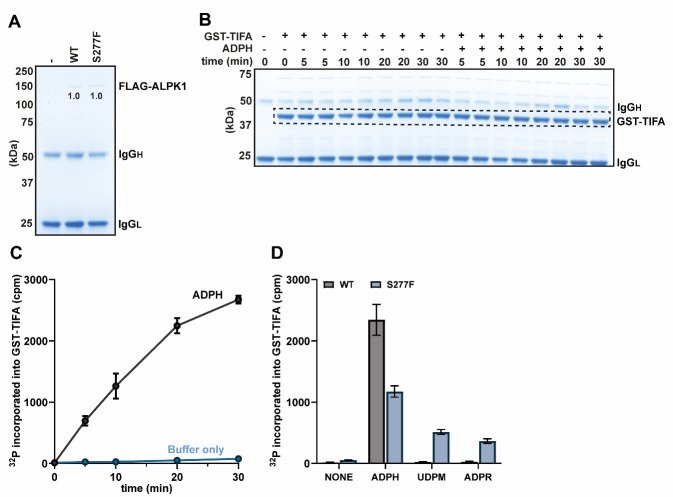
Phosphorylation of GST-TIFA by FLAG-ALPK1 and its mutants. (A)SDS-PAGE analysis showing the relative levels of ALPK1 in FLAG immunoprecipitations of the extracts prepared from ALPK1 KO HEK-Blue cells transfected with empty vector or plasmid encoding either FLAG-tagged WT ALPK1 or ALPK1[S277F]. The band intensities were quantified using ImageLab and were found to be identical in this experiment. **(B)** Stained SDS-PAGE gel from a time course phosphorylation experiment in the presence and absence of ADP-heptose (ADPH). The dashed box indicates the region corresponding to GST-TIFA, from which bands were excised for scintillation counting. **(C)** Quantification of the incorporation of radioactivity into GST-TIFA from the time course experiment shown in (B). **(D)** Phosphorylation assays were performed for 20 min with either WT ALPK1 or the ALPK1[S277F] mutant in the presence or absence of 10 µM ADP-heptose (ADPH), 100 µM UDP-mannose (UDPM), or 100 µM ADP-ribose (ADPR). For additional details, please see the main text. **(C, D)** The points in (C) and the bar heights in (D) represent the mean values, and the error bars indicate plus and minus one standard error of the mean.
  23. Prepare 0.5 mL aliquots of each cell extract on ice, which corresponds to 0.5 mg of WT FLAG-ALPK1 lysate and normalized amounts of lysate containing equivalent amounts of the respective mutant.
  24. There will typically be 15 aliquots of WT FLAG-ALPK1 from a 15 cm dish of transfected cells, which is sufficient for 150 kinase reactions. Snap-freeze these single-use aliquots and store them at -80 °C until use.
*Note: It is recommended to use these aliquots within 1 month and not re-freeze them once thawed, since the ALPK1 activity in the cell extract might be reduced or lost.*
- **Determination of linear rate conditions for WT ALPK1 (Days 4–5)**
  Kinase reactions must be performed under linear rate conditions, and it is therefore essential to perform a time course experiment with WT FLAG-ALPK1 for 5, 10, 20, and 30 min in both the presence and absence of ADP-heptose each time that cell extracts are prepared. This allows the optimal time at which to terminate the reaction to be determined for the experiments in Section D.
**Perform time course phosphorylation assays for WT FLAG-ALPK1:**
  1. The steps below describe how to assay WT FLAG-ALPK1 with and without ADP-heptose for 5, 10, 20, and 30 min, each time point performed in duplicate. A 0-min point is also included, performed without ADP-heptose (in singlet), plus an extra condition (in singlet) where GST-TIFA is not added. This is a total of 18 reactions (see Figure 1B for a summary of the 18 recommended conditions).
  2. Thaw two aliquots of WT FLAG-ALPK1 lysate on ice and combine them, sufficient for 20 reactions.
  3. Add 600 µL of anti-FLAG M2 affinity resin slurry (50% resin by volume) to a 1.5 mL microcentrifuge tube on ice, which is sufficient for 18 samples plus an excess of 10% (15 µL of resin per reaction).
  4. Centrifuge the slurry at 2,000× *g* for 1 min at 4 °C to pellet the resin. Aspirate the supernatant and resuspend the resin in 1 mL of ice-cold lysis buffer. Repeat the centrifugation and wash steps twice. After the final wash step, resuspend the dry resin in the lysate (1 mg) expressing WT FLAG-ALPK1. Incubate at 4 °C for 1 h on a rotating wheel.
  5. Wash the sample three times with ice-cold salt wash buffer, twice with ice-cold wash buffer, and once with ice-cold reaction buffer.
  6. Resuspend the 300 µL of dry resin in a total of 350 µL of ice-cold reaction buffer and aliquot 32.5 µL of this slurry into 18 individual 1.5 mL microcentrifuge tubes on ice, ensuring that the pipette tip is pushed to the bottom of the tubes to prevent the resin from drying out (i.e., do not pipette the resin onto the sides of the tubes). Each tube should now contain 15 µL of resin resuspended in 17.5 µL of reaction buffer, on ice.
  7. Add 2.5 µL of 1 mg/mL GST-TIFA (i.e., 2.5 µg, which is 2.1 µM) to each tube on ice (except the no GST-TIFA sample, where reaction buffer should be added instead), followed by either 2.5 µL of reaction buffer or 2.5 µL of 100 µM ADP-heptose in reaction buffer, on ice.
  8. Add 2.5 µL of the radioactive ATP solution (see Recipes) to the samples at 20 s intervals and put them onto a prewarmed thermomixer at 30 °C at 1,300 rpm, except for the 0 min timepoints, which should be terminated as described below, prior to the addition of the radioactive ATP solution.
*Note: The radioactive solution must be handled according to safety regulations.*
  9. To terminate reactions after the specified lengths of time have passed (i.e., 5, 10, 20, and 30 min), add 8.3 µL of LDS sample buffer 4× containing 10% (v/v) 2-mercaptethanol, heat for 5 min at 75 °C, and remove the resin using Spin-X columns.
*Note: Check the centrifuge and heat block for radioactive contamination.*
  10. Analyze half of the supernatant (i.e., 16.7 µL) by SDS-PAGE.
*Note: SDS-PAGE should be stopped before the dye front enters the running buffer, as this will minimize the generation of aqueous radioactive waste. The dye front should be excised from the gel and discarded as solid radioactive waste.*
  11. Stain with InstantBlue for 1 h and destain in water for 24 h with frequent changes.
*Note: Discard the destaining water as aqueous radioactive waste.*
Figure 1B shows the appearance of the stained gel but has been cropped above the 75 kDa marker, since the amount of FLAG-ALPK1 in the reaction is below the detection limit of the stain and therefore not visible.
**Quantify the incorporation of radioactivity into GST-TIFA:**
  12. Wash the SDS-PAGE gel five times in water for 5 min each to minimize background radiation.
*Note: Discard the destaining water as aqueous radioactive waste.*
  13. Transfer the SDS-PAGE gel to an A4 plastic pocket and dab with filter paper to remove excess water.
  14. Cut out individual bands corresponding to GST-TIFA (Figure 1B, dashed box) using a scalpel and transfer to 1.5 mL microcentrifuge tubes. Centrifuge the tubes containing the excised pieces of SDS-PAGE gel samples at 13,000× *g* for 1 min to bring the gel pieces to the bottom of each tube.
  15. Count each sample using a scintillation counter for 2 min per sample and plot the resulting data (Figure 1C).
  16. Count triplicate 1 µL aliquots of the radioactive ATP solution. Since the radioactive ATP solution contains 1 mM ATP (i.e., 1 nmol ATP per µL), the radioactivity of a 1 µL aliquot is the specific radioactivity in cpm per nmol of ATP. Knowing the specific radioactivity is necessary for comparing the results of different experiments and can be used to calculate the stoichiometry of phosphorylation (not shown).
  17. In this example, the 20 min timepoint was chosen for subsequent experiments because it was the longest timepoint that was still within the linear range of the assay (see Figure 1C).
- **Measurement of the activity of ALPK1 mutants in the presence of nucleotide sugars (Days 6–7)**
**Perform endpoint phosphorylation assays with different nucleotide sugars:**
  1. Thaw an aliquot of each cell extract on ice, sufficient for 10 reactions. Each lysate will be used for assays in the presence of buffer, ADP-heptose, UDP-mannose or ADP-ribose, each in duplicate, requiring eight reactions.
  2. Since there are two aliquots in total (WT and S277F), add 0.6 mL of anti-FLAG M2 affinity resin slurry (10% excess) to a 1.5 mL microcentrifuge tube on ice and centrifuge the slurry at 2,000× *g* for 1 min at 4 °C.
  3. Aspirate the supernatant and resuspend the resin in 1 mL of ice-cold lysis buffer. Repeat the centrifugation and wash steps twice.
  4. After the final wash step, resuspend the 300 µL of resin in 300 µL of lysis buffer and add 300 µL of this slurry to each of the aliquots. Incubate at 4 °C for 1 h on a rotating wheel.
  5. Wash each sample three times with ice-cold salt wash buffer, twice with ice-cold wash buffer, and once with ice-cold reaction buffer. After the final wash, resuspend the 150 µL of packed resin in each sample in a total volume of 175 µL of ice-cold reaction buffer.
  6. Aliquot 32.5 µL of each slurry into eight individual 1.5 mL microcentrifuge tubes on ice. Each tube should now contain 15 µL of resin resuspended in 17.5 µL of reaction buffer, on ice.
  7. Add 2.5 µL of 1 mg/mL GST-TIFA to each tube on ice, followed by either 2.5 µL of reaction buffer or 2.5 µL of 100 µM ADP-heptose, 1 mM UDP-mannose, or 1 mM ADP-ribose in reaction buffer on ice.
  8. Add 2.5 µL of the radioactive ATP solution (see Recipes) to the samples at 20 s intervals and put onto a prewarmed thermomixer at 30 °C at 1,300 rpm.
  9. Terminate reactions at the optimal timepoint determined in the preceding section by the addition of 8.3 µL of LDS sample buffer 4× containing 10% (v/v) 2-mercaptethanol and heat for 5 min at 75 °C.
  10. Remove the resin from the samples using Spin-X columns and analyze half of the supernatant (i.e., 16.7 µL) by SDS-PAGE followed by staining with InstantBlue for 1 h and destaining for 24 h in water with frequent changes.
**Scintillation counting to determine the incorporation of radioactivity into GST-TIFA:**
  11. Analyze the incorporation of radioactivity into GST-TIFA by scintillation counting using the same procedure described in Section C, steps 12–16. An example of the resulting data is shown (Figure 1D).

## Validation of protocol

This protocol or parts of it has been used and validated in the following research article(s):

- Snelling et al. [6]. Discovery and Functional analysis of a novel ALPK1 variant in ROSAH syndrome. bioRxiv (Figure 3, panels A and B; Figure 4, panel D)
- Snelling et al. [3]. ALPK1 mutants causing ROSAH syndrome or Spiradenoma are activated by human nucleotide sugars. Proc Natl Acad Sci USA (Figure 4, panels A–D)

## General notes and troubleshooting

**Troubleshooting**

- **Low or undetectable expression of ALPK1 constructs**
  This is most likely caused by a poor transfection efficiency, the study of a mutation that reduces the expression of ALPK1 (such as Tyr254Cys), or an issue with the immunoprecipitation procedure. To rule out the former, check the percentage of GFP-positive cells by flow cytometry or microscopy 24 h after transfection with a GFP plasmid such as pcDNA5-FRT/TO-GFP-ALPK1 (MRC PPU Reagents and Services, #DU78380). Below are general suggestions for troubleshooting.
  1. Ensure that cells are not overly confluent prior to plating into 15 cm dishes, as this can reduce the transfection efficiency and expression of ALPK1 constructs.
  2. Confirm that cells are not contaminated with mycoplasma or other infectious agents by routine testing.
  3. Check DNA purity by measuring the ratio of absorbance at 260 and 280 nm using a spectrophotometer, which should be between 1.8 and 2.0. Check DNA integrity by agarose gel electrophoresis and re-sequence.
  4. Analyze the expression of ALPK1 constructs in cell extracts by anti-FLAG immunoblotting, which will distinguish between failure to express and an issue with the immunoprecipitation procedure.
  5. Establish the efficiency of immunoprecipitation by comparing the level of ALPK1 in cell extracts before and after immunoprecipitation (i.e., input and supernatant) by immunoblotting.
- **Cell death during the transfection procedure**
  1. Ensure that cells are plated to be approximately 90% confluent at the time of transfection, as it is observed that cell toxicity from transfection increases as the confluency decreases below this value.
  2. Ensure that the culture media is replaced 4 h post-transfection, as this is observed to minimize cell toxicity.
- **High background phosphorylation signal**
  High background signal in immunoprecipitation-coupled phosphorylation assays can occur for numerous reasons, such as reactions not being within the linear range, contamination of buffers with microorganisms, inefficient washing following immunoprecipitation, or not rinsing the SDS-PAGE gel prior to excising bands. Below are general suggestions for troubleshooting a high background signal.
  1. Ensure that reactions are within the linear range of the assay by performing time course phosphorylation experiments in both the presence and absence of ADP-heptose, as described within the protocol.
  2. If high-level background radiation is observed in the absence of ADP-heptose, prepare fresh buffers to rule out contamination and confirm that ALPK1 KO HEK-Blue cells are not contaminated by carrying out routine testing (these may be sources of ADP-heptose in the reactions).
  3. Perform time course phosphorylation experiments from extracts expressing FLAG-ALPK1[K1067M] (MRC PPU Reagents and Services, #DU65680), which will establish whether the background signal is arising from the immunoprecipitation or ALPK1 itself, since this is a kinase-inactive form of ALPK1.
  4. Increase the number of washing steps with salt wash buffer to ensure that contaminating kinases are absent prior to performing phosphorylation reactions.
- **No or little phosphorylation of GST-TIFA in the assay**
  1. Confirm the integrity of GST-TIFA by SDS-PAGE and confirm that the concentration is correct.
  2. Ensure that the nucleotide sugars and GST-TIFA are in suitable buffers such as those described in the protocol. For example, EDTA present in buffers may inhibit phosphorylation reactions by chelating magnesium ions.
  3. Confirm whether ALPK1 is active by measuring the incorporation of radioactivity into ALPK1 itself (i.e., an autophosphorylation event). To do this, increase the amount of cell extract per reaction to 1 mg, as otherwise the amount of ALPK1 in the reaction will not be sufficient. As an example, see Figure 6C in [2].
  4. Ensure that protease inhibitors and DTT are included in the lysis buffer as described in the protocol and that all steps from cell lysis onward are performed on ice with ice-cold buffers unless stated otherwise in the protocol until the LDS sample buffer is added.
  5. Some ALPK1 mutants may be inherently unstable, which we observed in the case of Tyr254Cys [3]. It is recommended to perform experiments as quickly as possible after cell lysis and to minimize freeze-thaw.
